# Evaluation of insulin-like growth factor-I in postmenopausal women with breast cancer treated with raloxifene

**DOI:** 10.1186/1477-7800-4-18

**Published:** 2007-07-23

**Authors:** Benedito B da Silva, Daniel S Moita, Cleicilene G Pires, Edílson C Sousa-Junior, Alesse R dos Santos, Pedro V Lopes-Costa

**Affiliations:** 1 Department of Gynecology, Federal University of Piauí, Teresina, Piauí, Brazil

## Abstract

**Background:**

The objective of this study was to evaluate serum IGF-I levels in postmenopausal women with breast cancer treated primarily with raloxifene.

**Methods:**

Twenty-two postmenopausal patients with operable, stage I or II, estrogen receptor-positive carcinomas participated in this study. Following confirmation of diagnosis, the patients received 60 mg of raloxifene for 28 days prior to definitive surgery. Blood samples were collected for evaluation of serum IGF-I levels prior to initiating medication and following a 28-day treatment course. Student's t-test for paired samples was used in the statistical analysis. Significance was established at p < 0.05.

**Results:**

Mean serum IGF-I levels pre- and post-raloxifene treatment were 143.7 ± 9.7 ng/ml and 94.8 ± 7.6 ng/ml, respectively. This reduction in serum IGF-I levels following treatment with raloxifene was statistically significant (p < 0.001).

**Conclusion:**

Raloxifene significantly reduced serum IGF-I levels in postmenopausal women with breast cancer.

## Background

Insulin-like growth factor-I (IGF-I) is produced principally in the liver upon stimulation by GH and plays an important role in promoting normal and neoplastic cell proliferation [1-8]. Insulin-like growth factors I and II are almost exclusively bound to IGF binding proteins (IGFBPs), less than 1% circulating freely [7]. There are six types of IGFBP; however, more than 90% of all circulating IGF-I is bound to IGF binding protein-3 (IGFBP-3) [7,9,10]. IGFBP-3 inhibits the action of IGF-I at cell level by competitively binding IGF-I and thereby preventing it from binding to the IGF-I receptor [7].

The IGF system is currently recognized as a risk factor for the principal types of epithelial cancer [11,12]. Studies have shown an association of increased serum levels of IGF-I and decreased levels of IGFBP-3 with an increased risk of breast cancer in premenopausal women [13,14], suggesting that these patients may benefit from measures able to reduce serum IGF-I levels and increase IGFBP-3 levels. Nevertheless, a few studies have shown that some therapeutic strategies have succeeded in reducing serum IGF-I levels but without altering IGFBP-3 levels [2,7]. Steroidal and non-steroidal antiestrogens have been previously shown to inhibit the growth of estrogen receptor (ER)-positive cells, not only by acting as competitors of hormone agonists on nuclear receptors, but also by preventing the mitogenic action of the growth factor in the total absence of estrogens [15].

Significantly reduced serum IGF-I levels have also been reported in postmenopausal women with breast cancer treated primarily with tamoxifen [1,3]. However, tamoxifen exerts an estrogen-agonistic effect on the endometrium and when used for long periods of time increases the risk of endometrial carcinoma 3–4-fold in postmenopausal women [16], a fact that has triggered a search for alternative SERMS for the chemoprevention and treatment of breast cancer [17]. Raloxifene is a second-generation SERM that was initially approved by the US Food and Drug Administration for the prevention and treatment of osteoporosis; however, it was found to exert an antiestrogenic effect on the breast without stimulating the endometrium [18,19]. This fact was confirmed in the recently published Study of Tamoxifen and Raloxifene (STAR) trial, which showed that raloxifene is as effective as tamoxifen in reducing the risk of invasive breast cancer, as well as reducing the risk of endometrial carcinoma compared to tamoxifen [20].

To the best of our knowledge, only one study published in the literature has evaluated the effects of raloxifene, administered for a period of 14 days prior to surgery, on serum IGF-I levels as a primary treatment for breast cancer in postmenopausal women [2]. It is possible that women may benefit from the use of raloxifene over a longer period of time, both in chemoprevention and in the treatment of breast cancer. Therefore, in view of the paucity of reports in the literature on the primary effects of raloxifene on serum IGF-I levels in postmenopausal women with breast cancer, we decided to carry out the present study in which medication was administered for 28 days prior to definitive surgery.

## Patients and Methods

### Patients

The protocol of this study received the approval of the Institutional Review Board of the Federal University of Piauí. All volunteers gave their signed, informed consent prior to initiation of the study. Twenty-two postmenopausal women in amenorrhea for at least two years, who had sought medical care at the Mastology Department of the Federal University of Piauí and who had been diagnosed with operable, estrogen-receptor-positive, invasive ductal carcinoma for which they had received no prior treatment, were enrolled to this study. Following hematoxylin-eosin staining and confirmation of the diagnosis of invasive ductal carcinoma, the paraffin blocks containing the samples underwent histochemical analysis to evaluate estrogen receptor status. Tumors with nuclear staining measured semiquantitatively as high (>10% immunoreactive cells) were considered positive. Patients with endocrinopathies, those in use of hormonal medication or any other medication that could interfere with serum IGF-I levels were excluded from the study. Tumors ranged from 2 to 5 cm in size, stages I or II. Patients were aged 47 to 87 years (mean 63 years).

### Treatment

The patients received 60 mg of raloxifene/day for a period of 28 days prior to definitive surgery, starting at the time of confirmation of the diagnosis following tumor biopsy.

### Sample collections

For the analysis of serum IGF-I levels, two fasting blood samples were taken, one at baseline, i.e. prior to the initiation of raloxifene therapy, and the second after a 28-day course of the treatment. Plasma was separated by centrifugation and aliquots were stored at -20°C until assayed. All tests were carried out by a professional who was blinded with respect to patient identification.

### Assay method

For the analysis of the serum concentrations of insulin-like growth factor-I, the automated immunoassay analyzer, DPC Immulite 2000 (DPC Inc., Los Angeles, USA) was used, and methodology consisted of a solid-phase enzyme-labeled chemiluminescent immunometric assay. Analytic sensitivity was 20 ng/ml and the reference values varied according to age.

### Statistical methods

Comparison of the means of serum IGF-I levels measured prior to and after 28 days of raloxifene use was carried out using Student's t-test for paired samples. Statistical significance was established at p < 0.05.

## Results

**Figure 1:**
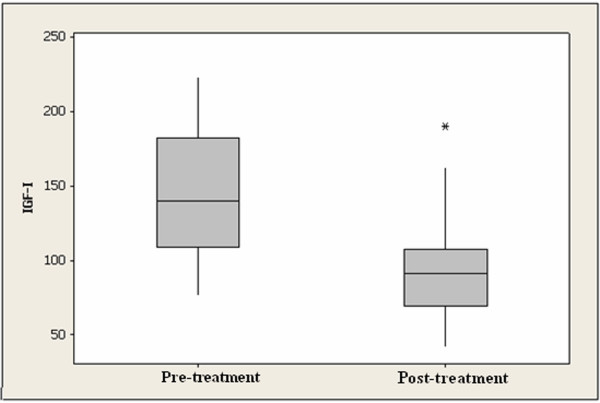
Boxplot of serum IGF-I levels measured prior to and following 28 days of raloxifene treatment in post-menopausal women with breast cancer.

Mean pretreatment serum IGF-I level was 143.7 ± 9.7 ng/ml, whereas mean post-treatment serum IGF-I level was 94.8 ± 7.6 ng/ml (Table 1). This difference was statistically significant (p < 0.001). The box-plot clearly shows a reduction in the median serum levels of IGF-I evaluated 28 days after the use of raloxifene in postmenopausal patients with breast cancer (Figure 1).

**Table 1:** Descriptive measurements of IGF-I measured prior to and following 28 days of raloxifene treatment

SerumIGF-I levels	Mean	S. E.	Minimum	Maximum
Before treatment	143.7	9.7	77.1	224.0
After treatment	94.8*	7.6	41.9	190.0

* The difference was statistically significant (p < 0.001).

## Discussion

Insulin-like growth factors are potent mitogens for the proliferation of breast cancer cells [1]. Insulin-like growth factor-I is a potent mitogen for breast cancer cell lines and it is now accepted that elevated IGF-I levels are a risk factor for breast cancer in the premenopause [3]. Therefore, women at risk of breast cancer or patients with neoplasia may benefit either preventively or therapeutically from strategies to reduce serum IGF-I levels [2]. SERMs have been shown to reduce both circulating and microenvironmental levels of IGF-I, thereby suppressing IGF-induced growth of both ER-positive and ER-negative breast cancer cells [1]. Nevertheless, the adverse effects of tamoxifen, principally stimulation of the endometrium, have evoked interest in studying other SERMS that may have a lesser negative effect or no negative effect on the endometrium and a similar or greater efficacy in chemoprevention or in the treatment of breast carcinoma [16,17].

In the present study, raloxifene, at a dose of 60 mg/day for 28 days, significantly reduced serum IGF-I levels in postmenopausal women with estrogen receptor-positive breast carcinoma. The schedule of 28 days of use of the medication by postmenopausal women prior to surgery was chosen with the intention of administering the drug for a longer period of time; however without delaying definitive surgery, 28 days being the mean time in our institute between the first consultation and surgery. In addition, the dose of 60 mg of raloxifene was chosen because it is the most commonly used dose for the prevention and treatment of osteoporosis and in clinical trials in the chemoprevention of breast cancer [19]. Serum levels of IGBP-3 were not evaluated; however some studies have shown no changes following the administration of raloxifene, except a decrease in the IGF-I/IGFBP-3 molar ratio following raloxifene treatment [2]. Our study was not placebo-controlled, and therefore subjects were not randomized to a treatment regimen. A placebo-controlled study would have had distinct design advantages over the study described here; however, controversies on the ethical implications of such trials have been the subject of recent collaborative reports [3,21].

Our findings of a reduction in serum IGF-I levels in postmenopausal women with breast cancer following treatment with raloxifene are in agreement with data published by other investigators [2]. The study of serum IGF-I levels only in women with estrogen receptor-positive tumors does not appear to be an issue since SERMs reduce serum IGF-I levels both in women with estrogen receptor-positive and negative tumors [1,22]. Apart from their main action via estrogen receptors, SERMs possess numerous other plausible mechanisms for controlling tumor growth, such as binding to protein kinase C and inhibiting angiogenesis [22,23]. An experimental study has shown angiogenesis inhibition in an estrogen receptor-negative animal model, suggesting that the antiangiogenic effects of SERMs are partially due to mechanisms that do not depend on estrogen receptors [24]. In addition, reports from other experimental studies have suggested the possibility that the action of antiestrogens does not occur only via estrogen receptors but also by direct inhibition of growth factors [22], which may explain the response of some estrogen receptor-negative breast tumors to SERMs.

Interest in the role of the insulin-like growth factor (IGF) axis in carcinogenesis has grown following the finding of elevated serum levels of insulin-like growth factor-I in association with the principal forms of epithelial cancer [9,12]. In addition, IGF-I is a systemic hormone with potent anti-apoptotic and mitogenic properties that may influence the proliferative behavior of breast cells [14]. Raloxifene has been shown to be effective in inhibiting cell proliferation both in normal and in neoplastic breast tissue [17]. According to one recent report, the drug significantly reduced Ki-67 antigen expression in the breast tissue of premenopausal women [17]. Finally, the reduction in serum IGF-I levels by raloxifene in postmenopausal women with breast cancer supports the need to conduct further clinical trials on adjuvant therapy and chemoprevention with raloxifene.
